# Predicting Future Reading Problems Based on Pre-reading Auditory Measures: A Longitudinal Study of Children with a Familial Risk of Dyslexia

**DOI:** 10.3389/fpsyg.2017.00124

**Published:** 2017-02-07

**Authors:** Jeremy M. Law, Maaike Vandermosten, Pol Ghesquière, Jan Wouters

**Affiliations:** ^1^Parenting and Special Education Research Unit, Faculty of Psychology and Educational Sciences, KU LeuvenLeuven, Belgium; ^2^Laboratory for Experimental ORL, Department of Neuroscience, KU LeuvenLeuven, Belgium

**Keywords:** rise-time discrimination, dyslexia, longitudinal studies, child development, frequency modulation

## Abstract

**Purpose:** This longitudinal study examines measures of temporal auditory processing in pre-reading children with a family risk of dyslexia. Specifically, it attempts to ascertain whether pre-reading auditory processing, speech perception, and phonological awareness (PA) reliably predict later literacy achievement. Additionally, this study retrospectively examines the presence of pre-reading auditory processing, speech perception, and PA impairments in children later found to be literacy impaired.

**Method:** Forty-four pre-reading children with and without a family risk of dyslexia were assessed at three time points (kindergarten, first, and second grade). Auditory processing measures of rise time (RT) discrimination and frequency modulation (FM) along with speech perception, PA, and various literacy tasks were assessed.

**Results:** Kindergarten RT uniquely contributed to growth in literacy in grades one and two, even after controlling for letter knowledge and PA. Highly significant concurrent and predictive correlations were observed with kindergarten RT significantly predicting first grade PA. Retrospective analysis demonstrated atypical performance in RT and PA at all three time points in children who later developed literacy impairments.

**Conclusions:** Although significant, kindergarten auditory processing contributions to later literacy growth lack the power to be considered as a single-cause predictor; thus results support temporal processing deficits' contribution within a multiple deficit model of dyslexia.

## Introduction

Dyslexia is a hereditary neurodevelopmental disorder characterized by persistent, lifelong reading, and/or spelling impairments that cannot be accounted for by low intelligence or environmental factors (Vellutino et al., 2004). Recent etiological views of dyslexia have proposed a multi-cognitive deficit model explaining the behavioral traits associated with this disorder (Pennington, 2006). It is theorized that multiple genetic or environmental factors act probabilistically as risk or protective factors. Thus, the interaction of these etiological factors result in the development of the specific cognitive risk or protective factors that increase or decrease the probability of the development of the expressed behavioral symptoms attributed to dyslexia.

One prominent etiological risk factor thought to be at the core of dyslexia, and found across all languages, is a deficit in the formation of, and/or access to, phonological representations (Snowling, 2000; Ramus and Szenkovits, 2008). As phonological skills have been shown to be vital in later literacy achievement, a disruption in the formation of phonological representations have negative consequences for literacy outcomes. For instance, pre-reading phonological awareness (PA) has shown to account for 40–60% of the later reading achievement of kindergarten children (Bryant et al., 1990; Torgesen et al., 1994; Caravolas et al., 2001). PA, which is the ability to recognize, isolate, and manipulate basic speech units, develops early in life, prior to reading instruction. It is believed that the awareness of larger segmental units of words, such as syllables, onsets, and rimes, develops first, while an awareness of smaller units, referred to as phonemic awareness, is thought to develop only after exposure to print (Goswami, 2002).

However, during the past few decades research has provided evidence suggesting that a more primary sensory deficit in dynamic auditory processing could be responsible for the observed phonological and literacy problems which underlie dyslexia (Tallal, 2004; Boets et al., 2007; Goswami, 2011; Steinbrink et al., 2014). It has been theorized that the underlying causes of phonological difficulties often observed in individuals with dyslexia stem from a deviant perception of specific temporal and dynamic auditory cues commonly represented in speech.

### Temporal auditory processing defects and dyslexia

Beginning with Tallal's 1980 study of the temporal order judgment of children with specific language impairments (SLI), research has explored the idea that the primary deficit of dyslexia could lay in deviant auditory processing skills.

Early research has related the interpretation of “temporal processing” strictly to rapid succession or short durational cues, as measured by gap detection tasks (Tallal, 1980). However, recent studies have demonstrated that the deficits observed in dyslexic readers are not mainly linked to the processing of short, rapidly presented stimuli, but especially to the processing of dynamic acoustic features such as frequency modulation (FM) and sound rise time (RT) (Goswami et al., 2002; Witton et al., 2002; Hämäläinen et al., 2005; Fraser et al., 2010; Boets et al., 2011; Poelmans et al., 2011; Law et al., 2014).

The processing of speech requires the interpretation and recognition of high-level perceptual units, such as words, sentences, and utterances. These perceptual units are an amalgam of various acoustic-phonetic cues that correspond to a time scale, specific to various phonological grain size units. For example, time windows of 0.14–0.33 s correspond to segmental information relating to syllable recognition, whereas phoneme identification relies upon the perception of shortened time scales of 0.02–0.08 s (Obrig et al., 2010). It is thought that during the pre-literate phase of development, a deficit in the perception, and processing of these acoustic-phonetic cues could ultimately limit a person's ability to isolate and reflect upon basal phonological information, thus resulting in inaccurate phonological representations (Nittrouer, 2006; Boets et al., 2007). This cascade of effects from a disruption in auditory processing, through speech, to the development of phonological representations has come to be known as the temporal auditory processing deficit theory (Ghesquière et al., 2014).

Supporting this theory, a growing body of research has provided evidence of a relationship between measures of dynamic auditory processing, phonology, and literacy achievement in pre-schoolers (e.g., Boets et al., 2011), in school-aged children (Talcott et al., 1999; Witton et al., 2002; Poelmans et al., 2011) as well as in adults (Hämäläinen et al., 2005; Law et al., 2014).

Speech perception research in the dyslexic population has primarily relied upon two experimental paradigms: (i) the perception of speech presented in back ground noise and (ii) categorical perception of stop consonant, often utilize optimal listening conditions, and do not involve whole word perception (for a review see Vandermosten et al., 2010). Past studies utilizing the more ecological speech-in-noise measure have demonstrated that children (Snowling et al., 1986; Wible et al., 2002; Bradlow et al., 2003; Ziegler and Goswami, 2005; Boets et al., 2011) and adults with dyslexia (Dole et al., 2012; yet see Hazan et al., 2009; Law et al., 2014) exhibit pronounced difficulty on speech perception tasks under noisy background conditions, while often not demonstrating any impairment of speech perception in silent conditions (Bradley and Bryant, 1983; Bradlow et al., 2003). Additional support for speech-in-noise deficits of individuals with dyslexia has been provided through neurophysiologic studies. Anomalies have been observed in the neural encoding of speech-in-noise stimuli of individuals with dyslexia when compared to normal reading controls (Wible et al., 2002), yet no differences were found between groups in quite listening conditions (Cunningham et al., 2001).

In accordance with the temporal auditory processing theory, we would expect measures of dynamic auditory processing to relate with performance on speech-in-noise perception measures. In an investigation of pre-reading children by Boets et al. (2011) a clear relationship between a measure of auditory processing and speech-in-noise perception was found. Results of this study demonstrated that children who went on to develop dyslexia were already impaired in dynamic FM sensitivity and speech-in-noise perception prior to reading instruction. These measures were also found to uniquely predict later growth in reading. Yet in two more recent studies no clear evidence was found supporting a relationship between dynamic auditory processing and speech-in-noise perception in kindergarten and 6th grade (Poelmans et al., 2011; Vanvooren et al., submitted).

### Measures of dynamic auditory processing

Two of the more sensitive dynamic auditory measures in differentiating between individuals with dyslexia and controls have been shown to be the FM and RT tasks. FM detection assesses an individual's ability to detect fluctuations in a carrier frequency at a certain modulation rate. Individuals with dyslexia have been shown to have a reduced sensitivity to FM detection when compared to control groups, demonstrating the ability of FM tasks to differentiate between adult, school aged and pre-reading dyslexics from normal readers (Witton et al., 1998, 2002; Ramus et al., 2003; Boets et al., 2007; yet see Halliday and Bishop, 2006; Stoodley et al., 2006; for a review see Hämäläinen et al., 2013). In addition to the findings of group differences, a study by Witton et al. (1998) found that the phonological decoding skills of both dyslexics and controls significantly correlated with FM sensitivity of 2 and 40 Hz.

More recently RT detection tasks, another measure of dynamic auditory processing, have been shown to be a more sensitive measure in discriminating between populations of dyslexic and normal readers. RT discrimination tasks measure an individual's ability to detect subtle differences in the rate of change of an amplitude envelope. RT tasks allow for an indirect assessment of how well an individual can detect the onset of syllables which are necessary for speech perception (Goswami et al., 2002; Goswami, 2011; Poelmans et al., 2011) and are utilized in the segmentation of the speech signal into its base parts, such as syllables, and onset/rime (Goswami et al., 2010). Detection of such cues has been shown to be associated with the reading, writing and the phonological skills of adult and child populations (Goswami et al., 2002; Hämäläinen et al., 2005; Thomson et al., 2006; Pasquini et al., 2007; Thomson and Goswami, 2008; Fraser et al., 2010; Goswami, 2011; Law et al., 2014, 2016). Goswami et al. (2002) demonstrated that 25% of the unique variance in the reading and spelling of children could be predicted by individual differences in RT sensitivity, when IQ and age are controlled for. Additionally, findings demonstrating the relationship between RT and reading have also remained consistent across different orthographies (Goswami, 2011).

### Criticisms of the temporal auditory processing deficit theory of dyslexia

Yet not all studies have been able to replicate support for the temporal auditory processing theory (Halliday and Bishop, 2006; Stoodley et al., 2006; White et al., 2006). Though theoretically appealing, the temporal auditory processing deficit theory of dyslexia has faced criticism regarding the use of adequate controls for the psychophysical tasks in addition to questions concerning directionality and the lack of a clear association between speech-in-noise perception tasks and auditory processing deficits.

Questions have been raised regarding the observed poor performance of individuals with dyslexia in psychophysical studies, in that such observations may be a function of a general difficulty with task completion, thus resulting in the misinterpretation of non-sensory difficulties, such as those with attention or general task difficulty, as sensory ones (Stuart et al., 2001; Roach et al., 2004). To address such concerns Poelmans et al. (2011) utilized an intensity discrimination (ID) task, matched in design and methodology to the other experimental dynamic auditory processing tasks to act as a control measure. The inclusion of an ID measure permitted Poelmans and colleagues to rule out related task demands, attention, and cognitive aspects as driving factors of the observed auditory problems. This is in line with the observation that group differences between typical (normal) and dyslexic readers are often not found in measures of ID (see Hämäläinen et al., 2013).

Additional criticism has been drawn regarding the directionality and causality of the proposed theory. Arguments have been put forth stating that the processing of basic auditory stimuli may be affected in a top-down manner through poorly specified phonemic representations and are a consequence of the poor reading experiences (Bishop et al., 2012). Evidence to support such a top-down relationship has been provided in two studies, suggesting that the auditory system gets tuned into listening for particular frequency and /or amplitude changes, during speech perception, thus creating a situation where the individual favors the processing of speech-specific auditory cues (Nittrouer and Miller, 1997; Mayo et al., 2003). For instance, both studies have demonstrated that mature cue weighting strategies for speech perception develop in childhood as a result of increasing phonological awareness. Yet, a study by Johnson et al. (2009) noted evidence of a bidirectional relationship of phonological awareness and auditory processing. As most studies have centered on a single time point and populations of adults and school aged children after the onset of literacy instruction (for a review see Hämäläinen et al., 2013), questions of directionality and causality are difficult to address. In one of the few studies which investigated pre-reading auditory processing deficits in children longitudinally and could provide evidence of directionality, Boets et al. (2011) retrospectively explored the temporal auditory processing deficit theory in a population of pre-reading Dutch speaking children who later developed dyslexia. Through a series of partial cross-lagged correlations Boets and colleagues could not support a reliable interpretation of directionality, leading to the conclusion of a probable bidirectional relationship between auditory processing, speech-in-noise perception, and phonological awareness. Supporting these findings a longitudinal study of pre-reading children by (Vanvooren et al., submitted) found no evidence for a unidirectional causal link between auditory processing, speech-in-noise perception and phonological awareness during the first stages of reading acquisition.

Lastly, the temporal auditory processing deficit theory of dyslexia has received criticism relating to the lack of a clear association between speech-in-noise perception tasks and auditory processing deficits in the literature, thereby calling into question the viability of the theory (Rosen, 2003). Although studies have demonstrated deficits independently in the dynamic processing and speech-in-noise perception in individuals with dyslexia, only a handful of studies have assessed measures of both in the same population (Boets et al., 2011; Poelmans et al., 2011; Law et al., 2014; Vanvooren et al., submitted). Using RT and FM discrimination measures Poelmans et al. (2011) examined the same population of children of the longitudinal study of Boets et al. (2011) in at the age of 11, and although a relationship among dynamic auditory processing and speech-in-noise perception was present at an earlier age (Boets et al., 2011), Poelmans et al. found no clear evidence supporting a relationship at a later age. Additionally, using similar measures, Law et al. (2014) was unable to support such a relationship in an adult population. Such results suggest that the observed auditory processing problems and their association with speech perception skills in individuals with dyslexia are present at birth through early childhood, thus contributing to early phonological deficits (Corriveau et al., 2010). However, auditory processing problems may diminish through development and eventually become resolved. The diminishing of the severity of the auditory impairment and its association with speech perception through time may obscure potential effects of this deficit in later reading achievement and related skills (Galaburda et al., 2006). Therefore, to fully understand the relationship of auditory processing and speech-in-noise perception early in development a replication of the findings pertaining to FM discrimination of Boets et al. (2011) and the inclusion of the more sensitive RT measure pre-reading is required.

### The present study

The aim of the current longitudinal study is to address the above criticism of the temporal auditory processing deficit theory and to offer evidence in support of the theorized cascade of effects from auditory processing through speech in noise perception to phonological awareness and ultimately disrupting reading. Although previously examined in other languages this study will attempt to replicate earlier findings in an English speaking, pre-reading population. In addition to the FM detection task used in Boets et al. (2011), the more sensitive measure of RT was added as an assessment of speech envelope cues and to reflect the growing body of evidence of the importance of such cues in the early development of phonological awareness (Corriveau et al., 2010). Similar to Poelmans et al. (2011), an ID task was included in the testing battery to act as a means of control for attention difficulties and task related demands.

The objectives of this paper are 3-fold. First, to determine the relation between the kindergarten measures of auditory processing and speech-in-noise perception tasks, and the cognitive and literacy outcome measures at grades 1 and 2. Secondly, this study will attempt to address the question of directionality through an examination of pre-reading RT discrimination, FM sensitivity and PA ability to reliably predict later literacy achievement. Lastly, we investigate the presence of performance differences between groups based on behaviourally observed literacy problems across three time points: pre-reading kindergarten, grade one, and grade two.

## Methods

### Participants

Fifty-eight preschool children ranging in age from 4 to 5 years old and attending Senior Kindergarten (SK) in the Ontario Canada public school system were originally selected for the study. At the completion of the third year of the study, 44 children remained. Three children were absent due to relocation to a school district not included in the study and one child's parents chose not to participate in the second phase of data collection. Additionally, to reduce the influence of second language learning on the sample, 10 children were removed from the study after enrolling in a French immersion education program. Children were initially recruited to meet one of two classifications, either being at high-risk (HR) for developing dyslexia, or being at low risk (LR). The high-risk group was selected based on the child having at least one first-degree family relative with an official diagnosis of dyslexia. The low-risk group consisted of children with no family history of reading difficulties. Groups were matched on measures of intelligence, socioeconomic status, gender, age, hyperactivity and educational environment (see Table 1). All participants were reported through parental surveys to possess no signs of brain damage or long term auditory or visual impairments and were native English speakers. Only children considered “pre-readers” were initially included in the study. For the purposes of this study a “pre-reader” was defined as a child who had not received any formal reading instruction prior to the first testing period. Additionally children who demonstrated ceiling performance on our measure of letter knowledge proficiency along with the ability to decode three consecutive words from the target word list of the *Wide Range Achievement Test* (WRAT3) (Snelbaker et al., 2001; e.g., red, milk, was) were excluded from the study.

**Table 1:** **Participant characteristics, groups passed on retrospective assignment**.

	**Control (*n* = 19)**	**DYS (*n* = 21)**	***p*-value**
Gender (F/M)	10/9	10/11	0.987^b^
Age in months (mean ± SD)	64.5 ± 4.2	62.1 ± 2.7	0.057^c^
Non-Verbal IQ^a^ (mean ± SD)	109.6 ± 7.0	106.5 ± 6.5	0.154^c^
Hyperactivity (mean ± SD)	2.7 ± 1.7	3.3 ± 2.1	0.337^c^
SES (ISCED) (low/middle/high)	1/10/9	1/13/7	0.838^d^
Mother's education (SE/PSE/GS)	3/12/4	4/14/3	0.843^d^
Father's education (SE/PSE/GS)	5/10/4	5/13/3	0.804^d^

DYS, Dyslexia group.

a Scores are standardized (M = 100, SD = 15).

b Pearson Chi-Square value.

c Independent-Samples t-test.

d *Fisher's Exact test. SE, secondary school education; PSE, post-secondary education; GS, graduate studies*.

Participation was voluntary. Upon registering parents completed an online questionnaire which informed the study of the child's medical history, behavior and family history of reading and spelling (dis)abilities. The parental questionnaire also included screening for potential hyperactivity or behavioral problems, using questions taken from the Strengths and Difficulties Questionnaire (SDQ) (Goodman, 2001). Additionally, parental educational levels were measured using the seven point ISCED-scale (UNESCO, 1997). Groups were found not to differ on measures of age, IQ, socioeconomic status (SES) and parental educational level, as can be seen in Table 1.

### Materials and procedures

#### Socio-economic status (SES)

Socio-economic status (SES) was assessed through the World Health Organization's (WHO) Family Affluence Scale II (FAS II). The FAS II is a four-part measure of family wealth scored as a composite measure ranging from 0 to 9. Similarly to Boyce et al. (2006) initial scores were transformed into Three categories of low affluence (0–2), middle affluence (3–5), and high affluence (6–9).

#### Intelligence (IQ)

The *Coloured Progressive Matrices* (Raven and Court, 1998) was utilized to assess the non-verbal intelligence of each child in kindergarten. The test consists of 36 items in Three sets measuring the spatial reasoning of participants. Each set within the test is arranged to measure the child's basic cognitive processes.

#### Literacy tests

Letter knowledge of the kindergarten group, including both receptive and productive letter knowledge, was assessed through the letter writing and naming subtests of the *Wide Range Achievement Test* (WRAT3) (Snelbaker et al., 2001). For each test the 15 most frequently occurring letters in English language books for children were used. In the situation where a child reached ceiling effect on letter knowledge the word portion of the WRAT3 was conducted. The reliability coefficient for this task was obtained utilizing the split-half method (Wilkinson, 1993) and found to be very high (0.98).

#### Reading

The word reading and word attack (a non-word reading test) subtest from the Woodcock-Johnson III was used to measure the reading and decoding skills of first and second grade children (Woodcock et al., 2001). Woodcock et al. (2001) reports a high reliability coefficient for this task (0.94). Standard procedures as outlined in the testing manual were followed during test administration. The word reading task consisted of 76 items while the non-word task contained 32 items. Each task progressively increased in difficulty. Scores were derived from grade based norms. Both measures were found to be significantly correlated (0.825 and 0.859 in grade 1 and grade 2 respectively). Thus, a composite was calculated from the mean of the z-scores from both word reading and non-word reading subtests.

#### Spelling

The spelling sub-test of the Woodcock-Johnson III (Woodcock et al., 2001) contained 59 target stimuli progressively increasing in difficulty was used to measure first and second grade spelling ability. Scores were derived from grade based norms. Standard procedural instructions as detailed in the Woodcock-Johnson III manual for administration and scoring were utilized. The reliability coefficient for this task was obtained utilizing the split-half method (Woodcock et al., 2001) and found to be high (0.92).

#### Phonological awareness (PA)

A subtest of the Clinical Evaluation of Language Fundamentals 4th edn (CELF-4) (Semel et al., 2003) was selected to assess each participant's phonological awareness ability at various grain size levels. The CELF-4 reports an overall internal consistency reliability coefficient alpha of 0.93. The subtest contains 11 parts of which seven were used: syllable blending (SB), 3 syllable deletion tasks (SD), syllable segmentation (SS), phoneme blending (PB), initial phoneme identification (IPI), medial phoneme identification (MPI), final phoneme identification (FPI). The PA score is based on the total score of all summed subtests. The syllable blending and 2 syllable deletion tasks were excluded from the calculation of PA for first and second grade students due to a high proportion of control subjects reaching ceiling effect.

#### Auditory processing tasks

All auditory tasks were conducted at the child's school and administered individually in a private room, free from distraction. All auditory tasks were controlled by APEX software (Laneau et al., 2005; Francart et al., 2008) on a Dell Latitude D510 computer. Auditory stimuli were presented through Sennheiser HDA 200 headphones to the right ear. All auditory processing task thresholds were estimated by means of a one-up, two-down adaptive staircase procedure which is designed to target a threshold corresponding to 70.7% correct responses (Levitt, 1971). Similar to Poelmans et al. (2011), all tasks were presented within a three-alternative forced-choice, “odd-one-out,” paradigm. Thus, in each trial the child was required to determine which of the three presented stimuli sounded different from the others. An inter-stimulus interval of 350 ms was used. All tasks were terminated after eight reversals. The arithmetic mean of the last four reversals was used as the threshold for each task. Each participant completed two threshold runs of each task, based on these scores test-retest reliability for each measure was calculated and is reported below. Due to our interest in threshold estimations as an indicator of an individual's sensory capability the best of these two runs was used as their threshold score (Boets et al., 2007, 2011; Poelmans et al., 2011; Law et al., 2014).

Two psychophysical threshold tests were used to assess temporal auditory processing. In the frequency modulation (FM) detection test, participants were required to detect a 2 Hz sinusoidal frequency modulation of a 1 kHz carrier tone with varying modulation depth. Modulation depth decreased by a factor of 1.2 from 100 to 11 Hz. At this point modulation depth decreases by a step size of 1 Hz. The reference stimuli was a pure-tone of 1 KHz. The duration of stimuli were 1000 ms including 50 ms cosine-gated onset and offset. The detection threshold was defined as the minimum depth of frequency deviation (in Hz) required to detect the modulation. The reliability coefficient for this task was obtained utilizing the test-retest method and found to be highly reliable (0.74).

The RT discrimination task consisted of a speech-weighted noise with linear amplitude rise times. Rise times varied logarithmically between 15 and 699 ms in 50 steps. The total duration of the stimuli was fixed to 800 ms, including a linear fall time of 75 ms. The reference stimuli of each trial was fixed at a 15 ms rise time. Discrimination thresholds were defined as the minimal difference in the RT required to discriminate between the reference and target stimulus. The reliability coefficient was obtained utilizing the test-retest method and found to be 0.72.

A non-temporal task, intensity discrimination (ID), was used as a control variable to correct for psychophysical task demands. The ID task was identical to the FM and RT discrimination tasks in its presentation and procedure. Participants were required to detect differences in intensity between a reference stimulus of 70 dB SPL and a target which varied linearly between 70 and 80 dB SPL in 40 steps of 0.25 dB SPL each. Discrimination thresholds were defined as the minimal intensity difference (in dB SPL) required to discriminate between the reference and the target stimulus. The reliability coefficient was obtained utilizing the test-retest method and found to be 0.54. A more detailed description of the stimuli can be found in (Law et al., 2016).

#### Speech-in-noise perception test

Words in noise perception was assessed with The Computer Aided Speech Perception Assessment (CASPA) developed by Boothroyd (2006) (for application see McCreery et al., 2010). A random selection of three lists of 10 CVC words were presented using the recording of a female speaker with a competing speech weighted noise at varying signal-to-noise ratios (SNR) (0, −5, and −10 dB). Each list contained a single occurrence of the same set of 30 phonemes (20 consonants and 10 vowels). A practice list of 0 dB SNR was first administered to the participant. Participants were instructed to repeat each target word or perceived phonemes after presentation. The percentage of correctly perceived phonemes was calculated for each SNR. The Speech Reception Threshold (SRT) was calculated for each participant through fitting to the data as a logistic function relating the percentage of correct responses to SNR level (for a similar approach see Poelmans et al., 2011). Final values for each measure were inverted by multiplying by a factor of −1 to obtain a positive correlation matrix.

### Statistical analyses

Statistical analyses were performed with SPSS 20.0 software (IBM Corp, 2011). Data from all variables were checked with Shapiro-Wilk's test for normality. All data were found to be normally distributed (*p* > 0.05) with the exception of some auditory processing data: FM and RT in kindergarten in addition to FM at both first and second grades as well as ID at first grade. In order to approach a normal distribution, variables were transformed by a logarithmic transformation. The assumption of homogeneity of variance was assessed by Levene's Test for Equality of Variances. Group comparisons were investigated based on an independent-samples *t*-test. Correction for multiple testing was applied across all group comparisons to avoid the likelihood of false positive conclusions through the application of the False Discovery Rate (FDR) procedure (Benjamini and Hochberg, 1995). The FDR procedure is a simple sequential Bonferroni-type procedure that has been demonstrated to control for the FDR for independent test statistics. Pearson correlations between kindergarten measures of auditory processing and speech perception tasks and cognitive measures at grades 1 and 2 were calculated. In addition Partial Pearson correlations with age, IQ and group as covariates were calculated.

To address the questions of directionality between the dynamic auditory processing measure of RT and PA a series of cross-lagged partial correlations while controlling for autoregressive effects, in addition to the effects of age, IQ and group, were performed. This method provides a way of drawing tentative causal conclusions regarding directional effects of auditory processing (Kenny, 1975).

In order to assess the predictive factors relating to first and second grade literacy measures (reading and spelling), four sets of simultaneous linear regression analyses were calculated across both groups. For each model later literacy performance in grade one and two was predicted by kindergarten measures of phonological awareness (PA), letter knowledge (LK), and dynamic auditory processing (RT) after controlling for Age and IQ.

## Results

### Relationship between early literacy, phonological awareness, auditory processing, and speech-in-noise perception

Table 2 shows concurrent and predictive relationships between all measures of dynamic auditory processing, speech-in-noise perception, phonological awareness, and measures of literacy.

**Table 2:** **Pearson correlations between kindergarten measures of auditory processing and speech perception tasks and cognitive measures at grades 1 and 2**.

**Kindergarten**						**Grade1**						**Grade 2**		
**Measure**	**1**	**2**	**3**	**4**	**5**	**6**	**7**	**8**	**9**	**10**	**11**	**12**	**13**	**14**
**KINDERGARTEN**														
1. RT	–	0.444^***^	0.217	0.337^*^	−0.027	0.609^***^	0.382^**^	0.123	0.466^**^	0.407^**^	0.211	0.442^**^	0.423^**^	0.281^∧^
2. FM	0.513^**^	–	0.086	0.111	0.095	0.373^*^	0.645^***^	0.101	0.151	0.045	0.000	0.048	0.038	0.119
3. SPIN	0.123	0.050	–	0.002	0.282^∧^	0.219	0.013	0.252	0.055	0.047	0.098	0.118	0.052	0.016
4. PA	0.197	0.192	0.144	–	0.519^***^	0.219	0.166	0.074	0.602^***^	0.604^***^	0.626^***^	0.672^***^	0.615^***^	0.583^***^
5. LK	0.116	0.017	0.402^*^	0.484^**^	–	0.046	0.154	0.078	0.298^*^	0.417^**^	0.507^***^	0.372^*^	0.403^**^	0.521^***^
**GRADE 1**														
6. RT	0.571^***^	0.405^**^	0.161	0.128	0.107	–	0.420^**^	0.100	0.410^**^	0.268^∧^	0.071	0.316^*^	0.281^∧^	0.162
7. FM	0.479^**^	0.628^***^	0.064	0.254	0.070	0.492^**^	–	0.109	0.293	0.165	0.113	0.137	0.150	0.027
8. SPIN	0.220	0.136	0.311	0.155	0.098	0.067	0.126	–	0.006	0.031	0.107	0.174	0.018	0.025
9. PA	0.473^**^	0.209	0.006	0.579^***^	0.204	0.412^**^	0.362^*^	0.034	–	0.709^***^	0.683^***^	0.798^***^	0.716^***^	0.629^***^
10. Reading	0.397^**^	0.083	0.083	0.545^***^	0.293^∧^	0.279^∧^	0.311^*^	0.037	0.676^***^	–	0.707^***^	0.867^***^	0.988^***^	0.871^***^
11. Spelling	0.191	0.040	0.166	0.627^***^	0.463^**^	0.048	0.141	0.245	0.625^***^	0.665^***^	–	0.716^***^	0.711^***^	0.719^***^
**GRADE 2**														
12. PA	0.406^**^	0.200	0.007	0.613^***^	0.216	0.304^*^	0.295^∧^	0.245	0.793^***^	0.804^***^	0.688^***^	–	0.863^***^	0.767^***^
13. Reading	0.421^**^	0.099	0.239	0.562^***^	0.270	0.299^∧^	0.300^∧^	0.021	0.690^***^	0.890^***^	0.674^***^	0.795^***^	–	0.864^***^
14. Spelling	0.261	0.005	0.134	0.551^***^	0.425^**^	0.160	0.143	0.007	0.575^***^	0.812^***^	0.686^***^	0.668^***^	0.801^***^	–

Lower left report partial correlations controlled for age, IQ and group.

∧ p < 0.07.

* p < 0.05.

** p < 0.01.

*** *p < 0.001*.

Of the two kindergarten dynamic auditory processing measures only RT correlated significantly with PA and the reading composite scores at all grade levels. Additionally, RT in first grade was found to be significantly correlated with PA, while it was found to be approaching significance with reading at grade one and two. However, speech-in-noise was not found to relate to any of the assessed measures across all time points. As would be expected from the auditory processing deficit theory both measures of auditory processing (RT and FM) were found to be significantly correlated within and between each grade level. However, auditory processing measures were not found to be related at any time point with measures of speech-in-noise.

When group, IQ, and age were introduced across all subjects to control for any spurious effects (see lower left half of Table 2) the majority of the relations were maintained with the exception of the relationship of kindergarten RT with Kindergarten PA, *r* = 0.197, *p* = 0.224; as well as the relationship of letter knowledge with grade 1 and 2 reading (*r* = 0.293, *p* = 0.067, and *r* = 0.270, *p* = 0.092).

**Figure 1:**
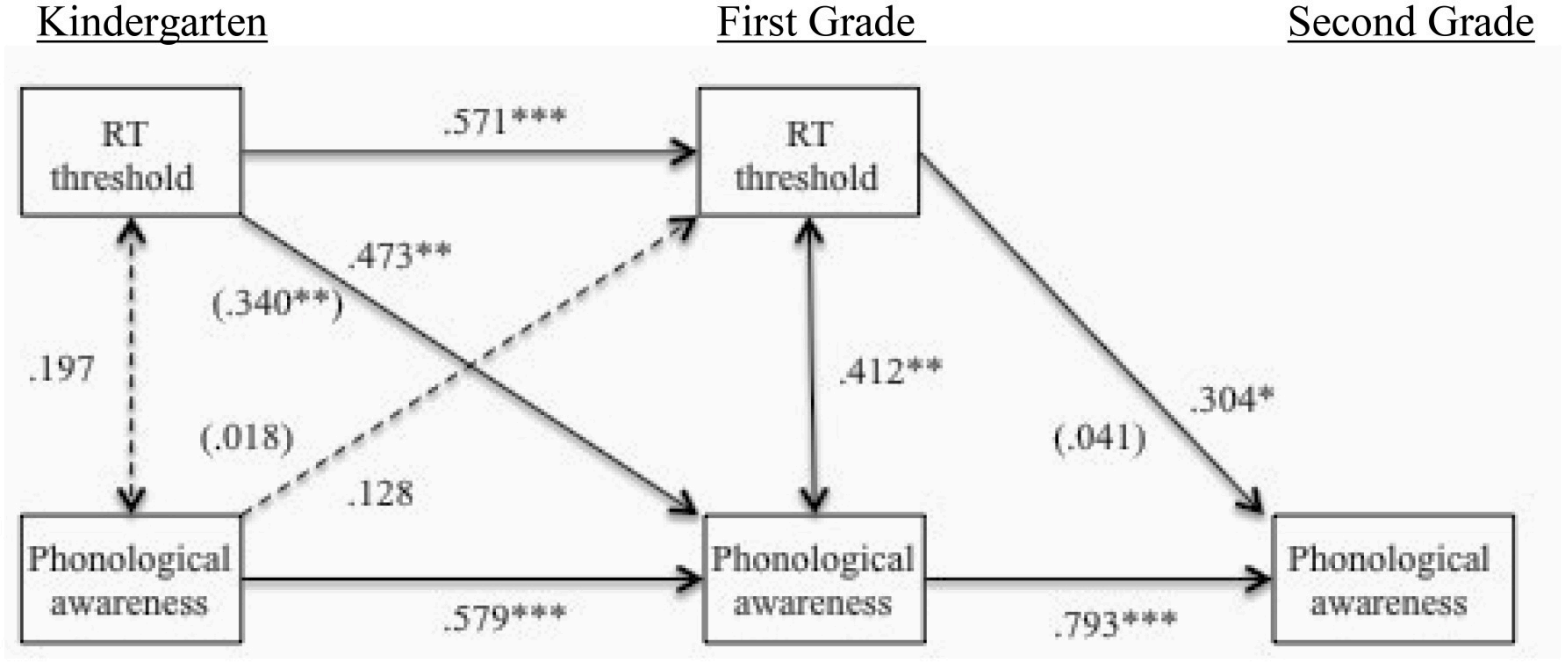
**Cross-lagged (partial) correlations modeling the relations between RT discrimination and phonological awareness across all time points, including covariates of IQ, age, and group**. Partial correlations corrected for autoregressive effect are presented in parentheses. ^*^p < 0.05. ^**^p < 0.01. ^***^p < 0.001.

Figure 1 displays concurrent, autoregressive and cross-lagged (partial) correlations. As FM was not found to significantly correlate with measures of PA, FM was excluded from this analysis. RT and PA in kindergarten and first grade were found to have a significant concurrent relationship. Significant predictive relationships of RT in kindergarten with first grade RT and PA measures were found and are depicted in Figure 1A. After controlling for autoregressive effects of kindergarten PA the predictive relationship of kindergarten RT and first grade PA was maintained, thus suggesting directionality.

### Predicting later literacy achievement by pre-reading RT discrimination, FM sensitivity, and phonological awareness

Results of the regression analysis shown in Table 3 revealed that phonological awareness, and the dynamic auditory processing measure of RT uniquely contributed to reading at both first and second grades. RT was found to account for 6.8% of the variance of first grade reading after controlling for PA, age, IQ, and letter knowledge, while PA was found to explain 9.5% of the variance after controls were accounted for. In the case of second grade reading after accounting for the variance of controls, RT accounted for 7.1% additional variance, in addition to the 11.0% of the variance attributed to PA. PA in kindergarten was found to explain an additional 13.4% of the variance of first grade spelling after accounting for controls. In addition, kindergarten PA was found to account for 7.5% of the variance of second grade spelling. At both time points letter knowledge was found to offer no significant contribution reading, yet was found to account for 6.2 and 6.3% of the variance of first and second grade spelling.

**Table 3:** **Unique variance in first and second grade reading, and spelling accounted for by kindergarten letter knowledge (LK), kindergarten phonological awareness (PA), and kindergarten rise time (RT) after controlling for age and IQ (***R***^**2**^change and standardized Beta)**.

**First grade**					**Second grade**			
	**Reading**		**Spelling**		**Reading**		**Spelling**	
	***R*^2^ change**	**β**	***R*^2^ change**	**β**	***R*^2^ change**	**β**	***R*^2^ change**	**β**
PA	0.099	0.404^*^	0.134	0.469^*^	0.110	0.425^**^	0.075	0.352^*^
LK	0.024	0.194	0.062	0.311^*^	0.017	0.166	0.063	0.316^*^
RT	0.068	0.292^*^	0.007	0.092	0.071	0.298^*^	0.027	0.184
Total *R*^2^	0.491		0.460		0.506		0.484	

* p < 0.050.

** p < 0.010. ^***^p < 0.001.

### Performance of children with dyslexia vs. non-literacy impaired readers

To investigate the presence of performance differences between groups based on the behaviourally observed literacy problems, the sample was retrospectively divided. Two groups, children with dyslexia and unimpaired children, were created based on their performance on literacy tasks at the start of second grade. A classification of literacy impairment was based on a child performing below the 10th percentile on two of the three second grade literacy measures: word reading, spelling or non-word reading. The resulting dyslexic (Dys) group consisted of 17 high-risk children and 4 low-risk children. The literacy unimpaired (control) sample was constructed of 19 low risk children. Four children from the high-risk group did not meet the cut-off criteria of dyslexia. Past research has demonstrated that similar groups of high risk normal reading children differ across many measures from low risk controls, so it was decided to exclude these individuals from group analysis of control subjects (Pennington and Lefly, 2001; Snowling et al., 2003). Additionally, due to the small sample size of high risk normal reading children separate statistical analysis was not performed on these subjects.

Tables 4–6 show the performance of children on all auditory, speech-in-noise perception, phonological awareness and literacy tests according to their classification and age. Independent *t*-tests found no group differences across measures of age, gender, IQ, SES, and hyperactivity (*p* > 0.05).

**Table 4:** **Performances on literacy, cognitive, auditory processing, and speech-in-noise perception tasks in kindergarten**.

	**Control**		**DYS**				
**Measure**	***M***	***SD***	***M***	***SD***	***t***	***p***	**Cohen's *d***
Letter Knowledge^∧^	0.3	0.3	−0.3	1.0	−2.444	0.022^*^	0.99
Phonological awareness	32.7	4.6	26.4	6.3	−3.535	0.001^*^	1.14
**AUDITORY TEMPORAL PROCESSING**							
Rise time (ms)	218.0	196.5	348.0	212.0	−2.385	0.022^*^	0.774
Frequency modulation (Hz)	10.6	8.9	9.7	9.7	−0.720	0.476	0.233
Intensity discrimination (dB)	3.2	1.3	3.7	1.5	1.072	0.291	0.348
Speech-in-noise (SRT) (dB)	−7.6	1.0	−7.7	1.3	−0.292	0.772	0.095

∧ failed Levene's test for Equality of Variance.

* *significant p-value after applying the FDR procedure to correct for multiple testing*.

**Table 5:** **Performances on literacy, cognitive, auditory processing and speech-in-noise perception tasks in grade 1**.

	**Control**		**DYS**				
**Measure**	***M***	***SD***	***M***	***SD***	***t***	***p***	**Cohen's *d***
**LITERACY**							
Reading	0.3	0.7	−2.2	0.9	−9.261	<0.001^*^	3.00
Spelling	112.2	10.5	100.7	6.7	−4.169	<0.001^*^	1.35
Phonological awareness	33.9	5.2	23.5	7.9	−4.870	<0.001^*^	1.58
**AUDITORY TEMPORAL PROCESSING**							
Rise Time (ms)	94.0	59.5	150.0	122.0	−2.165	0.037	0.702
Frequency modulation (Hz)^∧^	6.2	2.3	8.2	6.4	0.901	0.374	0.306
Intensity discrimination (dB)	1.9	0.8	2.5	0.9	1.890	0.066	0.613
Speech-in-noise (SRT) (dB)	−8.9	1.1	−8.9	1.7	0.125	0.901	0.040

∧ failed Levene's test for Equality of Variance.

* *significant p-value after applying the FDR procedure to correct for multiple testing*.

**Table 6:** **Performances on literacy, cognitive, auditory processing, and speech-in-noise perception tasks in Grade 2**.

	**Control**		**DYS**				
**Measure**	***M***	***SD***	***M***	***SD***	***t***	***p***	**Cohen's *d***
**LITERACY**							
Reading	0.9	0.5	−0.8	0.5	−10.462	<0.001^*^	3.39
Spelling	105.0	8.5	86.7	7.0	−7.471	<0.001^*^	2.42
Phonological awareness	39.2	2.8	29.6	5.9	−6.451	<0.001^*^	2.09
**AUDITORY TEMPORAL PROCESSING**							
Rise Time (ms)^∧^	73.0	46.5	125.0	136.5	−2.199	0.035	0.750
Frequency modulation (Hz)^∧^	5.4	2.7	6.7	9.7	0.853	0.399	0.277
Intensity discrimination (dB)	1.6	0.7	2.3	1.9	1.620	0.113	0.526
Speech-in-noise (SRT) (dB)	−10.1	1.6	−10.0	1.8	0.198	0.844	0.064

∧ failed Levene's test for Equality of Variance.

* *significant p-value after applying the FDR procedure to correct for multiple testing*.

#### Literacy and phonological awareness

Results of the literacy and phonological awareness tasks are found for all grades in Tables 4–6. Literacy in kindergarten was represented by a composite score formed by the averaging of z-scores of productive and receptive letter knowledge in kindergarten. Literacy in both first and second grade was measured by word reading, non-word reading, and spelling. Due to the highly significant correlation between word reading and non-word reading measures (0.825 and 0.859 in grade 1 and grade 2 respectively), a single reading score was created for each participant by averaging of z-scores of both tasks. Group comparisons, after the application of the FDR procedure revealed that dyslexic readers were found to perform significantly poorer than controls on all literacy measures in first and second grades. Group differences for letter knowledge were found to remain significant after the application of the FDR procedure.

Phonological awareness was assessed at both syllable and phoneme level. Independent sample *t*-tests, utilizing the FDR procedure, revealed significant differences between groups across at all time points (see Tables 4–6).

#### Auditory processing and speech-in-noise perception

As the aim of the auditory processing measures was to discover the threshold of the subject's sensory capability the best score of the two trials for each task was selected. Threshold means and standard deviations of all auditory stimuli at each grade level can be found in Tables 4–6. Group differences were not found for the control variable ID, thus assuring that group differences observed across the other auditory processing measures could not be attributed to task demands of the psychophysical tests and/or intensity-related processing.

Results demonstrated statistically significant poor performance of children with dyslexia on measures of RT discrimination at all three time points when a standard alpha of 0.05 was used: kindergarten [*t*_(38)_ = −2.385; *p* = 0.022], first grade [*t*_(38)_ = −2.165; *p* = 0.037] and second grade [*t*_(34, 396)_ = −2.199; *p* = 0.035]. Yet the same could not be said for measures of speech-in-noise perception, FM-detection nor ID. Although group differences were found for RT, significance was not maintained for RT at first and second grade time points after the application of the FDR procedure to correct for multiple testing.

## Discussion

In a longitudinal design this study set out to investigate the temporal auditory processing deficit theory, with a specific focus on dynamic auditory cues. This theory postulates that the primary deficit of dyslexia lays within poor auditory processing of speech specific auditory cues which cascades through speech perception disrupting the formation of phonological representations and ultimately impacting literacy achievement. Specifically this study sought to examine the directionality of these interrelationships and to determine whether future literacy achievements or difficulties could be predicted based on pre-reading dynamic auditory processing and speech-in-noise perception skills.

To achieve this end, a group of pre-reading children was followed from the start of kindergarten to second grade. Predictive relationships between pre-reading measures of auditory processing and emerging phonological and literacy skills were explored, and in addition, group differences for dynamic auditory processing, speech-in-noise perception and phonological measures were assessed based on the reading success or failure in second grade.

### Relations between speech-in-noise perception, auditory processing, and phonological awareness

Fitting with the auditory processing deficit theory of dyslexia, it was assumed that measures of speech-in-noise perception would be found to relate to both auditory perception and phonological measures. Yet this study was not able to demonstrate any evidence to support the existence of a speech-in-noise perception deficit in children with dyslexia. These results are contrary to past research (Snowling et al., 1986; Wible et al., 2002; Bradlow et al., 2003; Boets et al., 2007; Ziegler et al., 2009). In addition, the speech-in-noise measure was found to be unrelated to any of our measures of dynamic auditory processing, phonological awareness or literacy. Therefore, this study could not support the theorized directional pathway from auditory processing through speech-in-noise perception to phonological skills as proposed by the temporal auditory processing deficit theory. Three possible arguments can be made to explain these findings. Firstly, it could be argued that dynamic auditory processing either independently relates to reading measures or relates through phonological awareness and not through speech perception. However, this remains unlikely considering the prevalence of dynamic auditory cues in the speech signal. An alternative explanation offered by Poelmans et al. (2011) theorized that the developmental link between auditory processing and speech perception might diminish with age due to the effect of different developmental influences over time (also see the longitudinal study of Boets et al., 2011). Thus, the inability to discover a relationship between these measures may be a result of the age of assessemnt. At the age the children were assessed in our study, speech-in-noise perception not only relies on bottom-up auditory processing but also involves various top-down processes such as semantic and syntactic cues which may have masked the presence of a primary deficit. Past research has demonstrated the existence of a relationship between early auditory processing and later speech perception in infancy (Leppänen et al., 2010). In addition, it is known that a new-born's auditory processing is sensitive to all phonemic contrasts and quickly becomes constrained to acoustic features specific to their native language (Kuhl, 2004). Thus, auditory processing's influence on speech perception may be limited to the first year of life. As argued by Vanvooren et al. (submitted), impairment in the processing of speech specific auditory cues at a very early stage could potentially impede speech perception during early stages of language acquisition.

Nevertheless, a more plausible explanation of the lack of findings could be due to specific task characteristics. The lack of group differences most likely were a function of the stationary speech weighted background noise used as a speech mask. Dole et al. (2012) noted that such masking noises are less effective in differentiating between dyslexic and normal readers than modulated noises and background speech masks. Therefore, offering an explanation for some of the heterogeneity of findings surrounding speech-in-noise perception deficits of individuals with dyslexia across development (Hazan et al., 2009; see Boets et al., 2011; Dole et al., 2012; Law et al., 2014) and including the results reported here.

Yet it is important to consider that these results reflect only one aspect of speech perception, that being speech-in-noise perception. Although this measure does represent a more natural measure of speech perception it must be noted that this task relies not only on basic acoustic perception but also elements of auditory attention or selective attention which may have influenced the results.

Although RT discrimination and FM detection measures were not found to relate to the speech-in-noise measure, a significant relationship was found between these two pre-reading measures of dynamic auditory processing. In addition, kindergarten RT was found to relate to concurrent and later phonological awareness and reading in grades one and two. The findings of a pre-literate relationship of measures of RT and phonological awareness are in line with other longitudinal studies that explored RT and early pre-reading phonological awareness (Corriveau et al., 2010). Yet, kindergarten and first grade measures of FM were not found to relate to later phonological awareness. The lack of kindergarten FM's relationship with phonological measures contradicted findings by Boets et al. (2011) who found FM in kindergarten to correlate with measures of phonology across all grade levels. As the FM detection measure of this study closely mirrored that used by Boets and colleagues, a potential explanation of the inconsistent results could rely on differences in the phonological awareness measures used. As the PA measure of this study consisted of a greater proportion of phonemic awareness tasks then syllable or rime awareness. The grain size level of the PA measure is of importance when considering its relations with speech specific auditory processing measures such as FM. As discussed earlier, time windows of 0.14–0.33 s correspond to segmental information relating to syllable recognition, while phoneme segmentation is reliant on the perception of shortened time scales of 0.02–0.08 s (Obrig et al., 2010). As the stimuli used within the FM task was based on a 2 Hz sinusoidal frequency modulation, it would be reasonable to expect measures of FM to more closely relate with a PA measure assessing grain size units at the rime and syllable level, as demonstrated in Boets et al. (2011).

### Literacy achievement and pre-reading auditory processing and phonological awareness

Regression analyses of literacy measures accounting for letter knowledge and phonological awareness, and RT discrimination demonstrated kindergarten RT's ability to uniquely predict growth in reading achievement at grades one and two. Contrary to Boets et al. (2011), our dynamic auditory measure was found to uniquely predict variance in first and second grade reading suggesting that basic auditory processing skill's impact on reading development is not limited to the time point prior to reading instruction but extends through early stages of reading development. Results support the findings of Boets et al. (2011) in that individual differences in auditory processing are not simply a consequence of phonological awareness and early literacy achievement.

Although our results have demonstrated pre-reading RT measure's ability to predict later literacy skill, the variance explained within this model was limited supporting the findings of Plakas et al. (2013). Thus, highlighting auditory processing's role as one of many contributing risk factors in a multi deficit model of dyslexia, as theorized by Pennington (2006).

### Directionality of the hypothesised causal pathway

To address questions surrounding the directionality of the hypothesized causal pathway as predicted by the temporal auditory processing deficit theory, an investigation of the interrelations of auditory processing and phonological awareness across time points was conducted. Significant concurrent and predictive relationships were observed between the auditory processing measure of RT discrimination, and phonological awareness. Partial cross-lagged correlations, controlling for autoregressive effects, confirmed the directionality between dynamic auditory processing (specific to RT discrimination) and phonological awareness. Results demonstrated a larger impact of RT performance on future PA development than PA's influence on auditory processing development, thus supporting the bottom-up model proposed by Tallal (1980) within the first years of reading development.

Results contrasted with Boets et al. (2011) which demonstrated a lack of directionality between auditory processing (as measured through a FM discrimination task) and phonological awareness. It could be argued that RT sensitivity is less influenced by top-down processes during early stages of reading acquisition, and thus a more sensitive measure, when compared to FM, in establishing casual pathways as predicted by the theory.

### Performance of children with dyslexia vs. non-literacy impaired readers

In line with previous research (Pennington and Lefly, 2001; Snowling et al., 2003; Boets et al., 2011), children classified as dyslexic in grade two were found to differ significantly on all measures of phonological awareness, and literacy, across all three time points, when compared with typically developing readers.

As predicted by the temporal auditory processing deficit theory, group differences were expected across both measures of temporal auditory processing (RT and FM) but not for the non-temporal auditory ID control task. Group analyses demonstrated a statistically significant poorer performance of children later diagnosed with dyslexia on the measure of RT discrimination at the pre-reading phase, while a trend toward significance was observed for RT discrimination in first and second grade. Yet the same could not be said for measures of speech-in-noise perception, FM-detection or ID. The finding of poorer performance of children later found to be dyslexic on RT discrimination tasks prior to formal reading instruction indicates these problems are not consequential of the expressed literacy problems characteristic of dyslexia. These results were in line with the bulk of previous studies across age groups and languages (for a review see Hämäläinen et al., 2013).

The lack of significant group differences at each time point for the FM measure was unexpected as past research in both dyslexic children (Boets et al., 2011) and adults (Witton et al., 2002; Ramus et al., 2003) have demonstrated clear group differences. Similar to the results of this study, Law et al. (2014) unexpectedly reported a lack of group difference for FM in the presence of a RT-deficit. Law and colleagues suggested that such a difference in findings may imply the existence of a specific deficit in the perception of dynamic auditory cues related to the speech envelope, as measured through the RT discrimination task.

### Limitations

Several limitations regarding this work are worth noting. Although this study's sample size is comparable to the majority of the literature examining auditory processing and speech-in-noise perception in children with literacy impairments, the generalizability of the findings reported in this paper may be restricted due to the limited sample size of the study. Yet the validity of the conclusions of this study still remain valid because the group differences observed were confirmed by the correlational and regression analyses, i.e., pre-reading RT group difference is confirmed by unique predictive power on later reading achievement. Yet the restricted sample size did limit the statistical analysis we performed. A larger sample size would have permitted the use of structural equation modeling to allow for an analysis of the causal paths of the model we were investigating. It could be argued that sampling bias may have occurred during the recruitment. As enrolment for the study relied on parental responses to flyers sent home with children and did not involve a general sample, it could be argued that educationally motivated parents or parents concerned about their child's literacy success may had been more inclined to respond. The avoidance of this potential sampling bias was not possible due to restrictions placed on the solicitation of parent involvement by the school administration.

### Conclusion

Results were not able to directly support the proposed cascade of effects as predicted by the temporal auditory processing deficit theory. Yet, dynamic temporal auditory processing was found to uniquely predict a proportion of later literacy achievement. Thus, extending the power of predicting future literacy outcomes to developmentally earlier precursors. Yet, this proposed deficit model was incapable of entirely explaining all of the expressed behavioral traits observed in a dyslexic population. Our findings taken together with past research that has demonstrated that not all individuals with auditory processing or phonological impairments develop dyslexia (see Snowling, 2008; Boets et al., 2011) support the proposed multiple deficit model of Pennington (2006) which stresses the need to explore a multifactorial etiology which accounts for multiple risk or protective factors. Thus, through an investigation of alternative cognitive factors, such as orthographic or morphological processing (Bekebrede et al., 2009; Law et al., 2015), alternative perceptual factors (Stein, 2001) and biological explanations (Nicolson et al., 2001), the variance and comorbid symptoms associated with the dyslexic population can be better understood.

## Ethics statement

This study has been approved by the KULeuven Research Ethics Committee, and the Research Ethics Board at Nipissing University. Written informed consent was obtained from all parents and/or guardians of each participating child included in the study.

## Author contributions

JL was the main author of this paper, in addition was responsible for the organization and management of data collection and analysis. MV offered support in the editing and writing of the paper, as well as the data analysis of the paper. Both PG and JW were the primary supervisors of the study who provided financial and logistic support as well as training on administration of the tasks. Both had input in the direction and structure of the paper and provided support in the editing and data analysis of the paper.

### Conflict of interest statement

The authors declare that the research was conducted in the absence of any commercial or financial relationships that could be construed as a potential conflict of interest.
